# Primary Cutaneous B-cell Lymphoma Presenting in an Adolescent Male: A Case Report

**DOI:** 10.7759/cureus.90374

**Published:** 2025-08-18

**Authors:** Jana Alawadhi, Manar Alsaqabi

**Affiliations:** 1 Dermatology, Al-Amiri Hospital, Kuwait City, KWT

**Keywords:** case report dermatology, clinical dermatology, medical dermatology, pediatric dermatology, primary cutaneous b-cell lymphoma, skin nodule

## Abstract

Primary cutaneous marginal zone lymphoma (PCMZL) is a low-grade B-cell lymphoma of the skin that arises from mucosa-associated lymphoid tissue (MALT) and is characterized by a heterogeneous infiltrate of marginal zone B cells, small lymphocytes, and plasma cells. PCMZL is a subtype of primary cutaneous B-cell lymphoma (pcBCL). Pediatric PCMZL is exceedingly rare, with only a limited number of cases reported, and it is rarely encountered in clinical practice. Clinically, it typically presents in children or adolescents as solitary or multifocal skin lesions, most commonly on the limbs and trunk, with occasional involvement of the face. Histopathologically, the disease is marked by a mixed inflammatory infiltrate involving B cells and reactive immune cells. In children, PCMZL usually follows a slow-growing course, and treatment often involves localized or conservative therapies, with an overall excellent prognosis. Here, we report the case of a 16-year-old male patient presenting with a solitary skin nodule on the upper left arm.

## Introduction

Primary cutaneous B-cell lymphoma (pcBCL) is a heterogeneous group of lymphoproliferative disorders characterized by monoclonal proliferation of B lymphocytes primarily involving the skin [1-4]. There are three recognized subtypes: primary cutaneous marginal zone lymphoma, primary cutaneous follicle center lymphoma, and primary cutaneous diffuse large B-cell lymphoma (PCMZL) [1,4,5]. PCMZL is an indolent form of cutaneous B-cell lymphoma with origins in mucosa-associated lymphoid tissue (MALT) [5,6]. While pcBCLs account for approximately 20-25% of all primary cutaneous lymphomas in adults, with the median age of diagnosis being 55 years, their incidence in the pediatric population remains exceedingly rare [2-4,6-8].

Features of PCMZL usually present as asymptomatic solitary or multifocal red-brown papules and plaques with a predilection for the trunk and upper extremities [4,6,7]. The etiology of PCMZL remains unclear, but it is theorized to arise either as a primary neoplastic process or as a reactive proliferation driven by exogenous or endogenous stimuli. A small subset of cases has been associated with infectious agents such as *Borrelia burgdorferi* and hepatitis C virus [4-6]. The prognosis of PCMZL is promising, with a five-year survival rate of 93-99% in the majority of cases [4,5,7,8]. Given its rarity in the pediatric population, recognizing pediatric PCMZL is clinically important to avoid misdiagnosis or delayed diagnosis, which can lead to unnecessary anxiety, extensive workups, or inappropriate treatments.

The histopathology of PCMZL reveals a dense lymphocytic infiltrate, primarily distributed in the reticular dermis and often extending into the subcutaneous tissue. This infiltrate is described as nodular to diffuse in pattern and composed of marginal zone B cells, small lymphocytes, lymphoplasmacytoid cells, and plasma cells, frequently admixed with larger centroblast- or immunoblast-like cells and reactive T cells. The marginal zone B cells are typically small to medium in size, with irregular nuclei, inconspicuous nucleoli, and abundant pale cytoplasm [4-9]. Immunohistochemistry typically demonstrates expression of B-cell markers such as CD20 and BCL2, while lacking expression of CD5, CD10, and BCL6. These features help differentiate PCMZL from other cutaneous or systemic B-cell lymphomas [4,5,7,9,10].

## Case presentation

**Figure 1:**
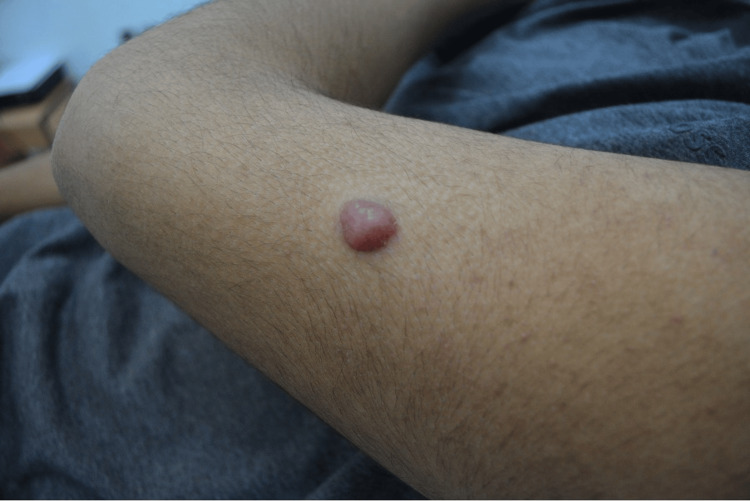
A firm, well-circumscribed, solitary 1 cm pink-red skin nodule on the upper left arm

A 16-year-old male presented at the dermatology clinic with an asymptomatic skin nodule on his upper left arm. The patient reports that the tumor had been increasing in size for one year before he decided to come to the clinic. Upon examination, it revealed a firm, well-circumscribed, solitary 1 cm pink-red skin nodule with the surrounding skin spared. The surface of the nodule is smooth and non-ulcerated (Figure 1).

**Figure 2:**
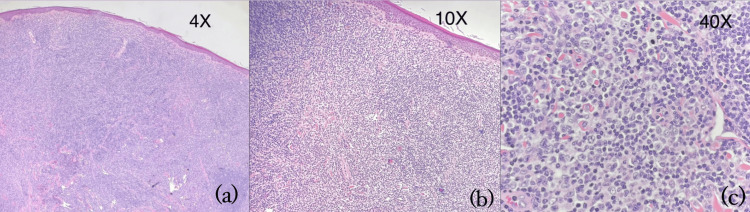
Histopathology of skin lesion (H&E stain) showing features consistent with primary cutaneous B-cell lymphoma (a) Diffuse infiltrate of atypical small- to medium-sized lymphoid cells; (b) background shows reactive lymphoid cells, plasma cells, and histiocytes with areas showing active germinal centers; (c) atypical lymphoid cells with moderate eosinophilic cytoplasm, hyperchromatic nuclei, and inconspicuous nucleoli H&E: hematoxylin and eosin

A systemic evaluation, including blood work, physical examination, and assessment of infectious causes, was conducted and came back as unremarkable. An excisional biopsy was then performed to help confirm the diagnosis. As shown in Figure 2, histopathology revealed a diffuse infiltrate of atypical small- to medium-sized lymphoid cells with moderate eosinophilic cytoplasm, hyperchromatic nuclei, and inconspicuous nucleoli. The background showed reactive lymphoid cells, plasma cells, and histiocytes. These features are consistent with cutaneous lymphoproliferative disorder, suggestive of PCMZL.

**Figure 3:**
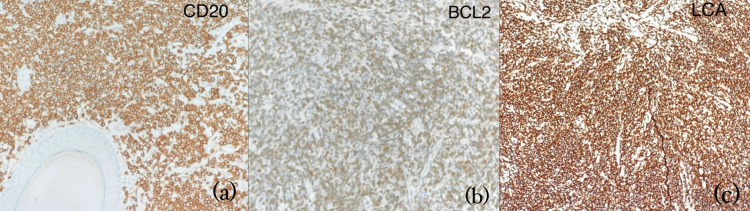
Immunohistochemistry of the skin lesion showing positivity for atypical lymphoid cell markers, consistent with a diagnosis of cutaneous B-cell lymphoma (PCMZL) (a) CD20 immunostain highlights atypical B-cell populations; (b) BCL2 shows positive staining in atypical lymphoid cells; (c) LCA positivity confirms lymphoid origin of the infiltrate PCMZL: primary cutaneous marginal zone lymphoma; LCA: leukocyte common antigen

As shown in Figure 3, immunohistochemistry revealed that the atypical lymphocytes were positive for LCA (leukocyte common antigen), CD20, and BCL2, consistent with a B-cell lineage. They were negative for CD3, CD5, CD10, BCL6, and ALK1, helping to exclude T-cell lymphomas, follicle center lymphoma, and systemic B-cell lymphomas. Background plasma cells were highlighted by CD38 and CD138 and demonstrated kappa light chain restriction, indicating clonality. Scattered immunoblasts stained with CD30 and background T cells were positive for CD3, CD4, and CD5 and weakly for CD7. The reactive germinal centers were positive for CD20, CD10, and BCL6 but negative for BCL2, further supporting a reactive process rather than follicular lymphoma. The Ki-67 proliferation index was approximately 20%, consistent with a low-grade lymphoproliferative disorder. These findings, along with the histological pattern and clinical presentation, support a diagnosis of PCMZL. In this case, the excision of the lesion served both as a diagnostic and therapeutic measure, and no further treatments were required at the time of follow-up.

## Discussion

PCMZL is a rare, indolent B-cell lymphoma of the skin arising from MALT [1-6]. While PCMZL is well-documented in the adult population, its occurrence in children and adolescents remains exceptionally uncommon, with limited studies documented and published [5,6]. In this case, a 16-year-old male presented with a solitary, asymptomatic pink-red nodule on the left upper arm, which is a clinical presentation consistent with previously reported pediatric cases. The slow progression over one year and the absence of systemic symptoms reflect the typically indolent course of PCMZL [4,6,7].

Histologically, the lesion demonstrated key features of PCMZL, including a diffuse dermal infiltrate of small- to medium-sized lymphoid cells with hyperchromatic nuclei and inconspicuous nucleoli, accompanied by plasma cells, reactive lymphoid cells, and histiocytes. These findings support a diagnosis of cutaneous lymphoproliferative disorder in keeping with PCMZL. The presence of a polymorphous lymphoid infiltrate and the lack of epidermal involvement are characteristic histologic patterns in PCMZL [4-7,9]. Immunohistochemistry typically reveals B-cell markers (e.g., CD20, BCL2), with negativity for markers such as CD5, CD10, and BCL6, which helps distinguish PCMZL from other cutaneous or systemic B-cell lymphomas [4,5,7,9,10].

The etiology of PCMZL remains uncertain, with hypotheses including both primary neoplastic transformation and reactive proliferation in response to chronic antigenic stimulation. Infectious triggers such as *Borrelia burgdorferi* and hepatitis C virus have been implicated in a minority of cases as well [4-6].

Management of pediatric PCMZL typically favors conservative approaches, given the disease's excellent prognosis and slow progression [4,5,7,8]. Therapeutic strategies may include observation or local treatments such as excision or intralesional steroids. Radiotherapy is generally avoided in pediatric patients due to long-term toxicity risks [2,4,5,7]. In this case, excisional biopsy served both diagnostic and therapeutic purposes, and no further intervention was necessary at the time of writing.

## Conclusions

This case highlights a rare case of PCMZL in an adolescent male, reinforcing the importance of including PCMZL in the differential diagnosis of persistent, solitary dermal nodules in pediatric patients. Despite its rarity, PCMZL follows an indolent clinical course with an excellent prognosis, and diagnosis is clinical and histopathologic. Early recognition and conservative management can lead to successful outcomes while minimizing unnecessary treatment-associated morbidity in pediatric patients. However, regular follow-up remains essential, even in indolent presentations, to monitor for potential recurrence or progression and to ensure timely intervention if needed.
